# Inaugural Meeting of the Cysticercosis Working Group in Europe^1^

**DOI:** 10.3201/1412.080889

**Published:** 2008-12

**Authors:** A. Lee Willingham, Leslie J. Harrison, Eric M. Fèvre, Michael E. Parkhouse

**Affiliations:** University of Copenhagen, Frederiksberg, Denmark (A.L. Willingham III); University of Edinburgh, Edinburgh, Scotland, UK (L.J. Harrison, E.M. Fèvre); Instituto Gulbenkian de Ciência, Oieras, Portugal (M.E. Parkhouse)

The first meeting of the Cysticercosis Working Group in Europe was held March 11–12, 2008, at the Instituto Gulbenkian de Ciência (www.igc.gulbenkian.pt), in Oeiras, Portugal, with organizational support from the World Health Organization (WHO)/Food and Agricultural Organization of the United Nations (FAO) Collaborating Centre for Parasitic Zoonoses in Denmark and the University of Edinburgh, Scotland. To exchange information about their various activities, the meeting brought together representatives from various European-based institutes and organizations involved in cysticercosis research and control efforts. The meeting was well attended by scientists actively involved with cysticercosis investigations in Europe, particularly in Spain and Portugal.The information provided by the 18 oral presentations served as a basis for discussion sessions aimed at finding ways to achieve a more effective, concerted approach to combating cysticercosis in Europe as well as in the main cysticercosis-endemic areas of Africa, Asia, and Latin America.

The meeting opened with an overarching situation analysis, followed by more specific presentations and then reports of 3 working groups. Lee Willingham emphasized that cysticercosis is a neglected disease and that it causes disabilities, interferes with work capacity, results in stigma and ostracism, and affects economically disadvantaged populations in remote areas. He provided illustrations of a range of current global efforts aiming to control cysticercosis and developments in methods to assess the disease distribution, prevalence, incidence, and societal impact, including economic costs. Demand for pork is growing consistently in developing countries, and this demand is met mainly by farmers with smallholdings, who do not or cannot prioritize disease control. A Global Campaign for Combating Cysticercosis is being organized to act as a driving force and help establish and support regional working groups to undertake activities based on regional needs and priorities. François Meslin echoed these issues and presented the problem of cysticercosis and opportunities for its prevention and control in the context of the new WHO-led initiative on the Integrated Control of Neglected Zoonotic Diseases (www.who.int/zoonoses/control_neglected_zoonoses/en/index.html). He stressed the challenges in developing intersectoral collaboration for control of neglected zoonotic diseases and emphasized the integrated “One Health” approach (www.who.int/entity/zoonoses/Report_Sept06.pdf). Cysticercosis and the other neglected zoonotic diseases are included as a subset of the neglected tropical diseases under WHO’s global plan to combat neglected tropical diseases during 2008–2015 (http://whqlibdoc.who.int/hq/2007/WHO_CDS_NTD_2007.3_eng.pdf).

Zbiginiew Pawlowski emphasized that *Taenia solium* cysticercosis/taeniasis is a focal disease and that it can be present at high prevalence in one location and very rare in a neighboring location. Much remains to be done in understanding this spatial variation. From a population perspective, an efficient way to deal with the human reservoir of infection, i.e., mass drug treatment of human tapeworm carriers as is currently undertaken for other neglected parasitic diseases such as schistosomiasis and soil transmitted helminths, although the effects of mass treatment on patients with nonsymptomatic neurocysticercosis would need to be considered.

Manuela Vilhena described the situation in Portugal. In parts of this country, cysticercosis transmission is most likely driven by the immigration from outside Europe of already infected patients of working age; in northern Portugal, evidence exists of transmission between humans and pigs in smallholder production systems.

Teresa Gárate showed that cysticercosis is rare in Spain but is an emerging problem among immigrants, primarily those from Latin America; 10% of the Spanish population originated outside Spain. The Institute of Health Carlos III in Madrid has developed highly sensitive and specific PCR-based assays for diagnosing *T. solium* infections as well as identifying *T. solium* antigens of diagnostic and protective potential.

A range of projects are under way in disease-endemic countries to study taeniasis, cysticercosis, and nonsymptomatic neurocysticercosis in humans with the support of European-based researchers, institutes, and agencies. In Tanzania, Erlich Schmutzhard, Joachim Blocher, and Andrea Winkler have, in collaboration with local counterparts, been investigating epilepsy and nonsymptomatic neurocysticercosis in the northern highlands. This research has been enabled by the presence of a new computed tomography scanner (the accepted standard diagnostic procedure); 14% of persons with epilepsy (compared with 2% of controls) had scans that were definitive or highly suggestive of nonsymptomatic neurocysticercosis. Lorraine Michelet, on behalf of Pierre Marie Preux, informed the group about France-funded research activities investigating the association between epilepsy and nonsymptomatic neurocysticercosis in several countries, the phylogenetics of *T. solium* isolates, and the development of molecular diagnostic tests for cerebrospinal fluid. Lee Willingham reported on the Denmark-funded project, Cross-Disciplinary Risk Assessment of *Taenia solium* Cysticercosis in Eastern and Southern Africa, which is promoting a comprehensive approach to porcine and human cysticercosis/taeniasis currently in Tanzania and Mozambique.

Several studies on porcine cysticercosis, smallholder pig production, and neglected zoonoses are also under way globally. Niels Kyvsgaard presented work from Nicaragua on scavenging versus restricted pigs, highlighting the point that for many smallholders, pig keeping is a low-input and economically viable activity. Vincent Porphyre described France’s support for research and development in pig farming and pork subsectors in tropical regions, including information sharing through a new website devoted to tropical pig production (http://pigtrop.cirad.fr). Paulo Duarte noted that the International Livestock Research Institute has recently received funding from the government of Portugal to conduct livestock research, including studies on porcine cysticercosis in Mozambique. Esther Schelling discussed Switzerland’s involvement in economic impact assessments of cysticercosis and cost-benefit assessments of potential prevention and control strategies. Mark Eisler outlined a project proposed to the European Commission aimed at investigating the presence, effects, and integrated control of a range of neglected zoonotic diseases (including cysticercosis) in sub-Saharan Africa.

Several European institutes are involved in developing immunologic and molecular diagnostic techniques for cysticercosis and taeniasis detection. Michael Parkhouse and Leslie Harrison presented work from the United Kingdom/Spain/Portugal collaborative research program on the use of the secreted metacestode glycoprotein HP10 to monitor treatment of severe neurocysticercosis and on the development of vaccines with the HP6 oncosphere adhesion molecule. Nynke Deckers reported that Belgium is currently supporting multiple epidemiologic studies in Africa, Asia, and Latin America; a *T. solium* vaccine trial in northern Cameroon; and the development of improved ELISA tests for cysticercosis as well as a PCR–restriction fragment length polymorphism test for taeniasis. Johan Lindh described Sweden’s efforts on diagnostics and vaccines in Nicaragua and Mozambique that primarily focused on identifying new immunogenic antigens from *T. solium* oncospheres. Patty Wilkins discussed the Centers for Disease Control and Prevention’s (CDC’s) antibody test development (immunoblot) for porcine and human cysticercosis and blood/stool tests for taeniasis. Currently, CDC is working on optimizing the format, sensitivity, and specificity of these tests with the goal of producing a dual serologic test that uses recombinant antigens. Gilbert Domingue illustrated the approach of the recently formed, nonprofit Global Alliance for Livestock Veterinary Medicines (www.galvmed.org) that aims to help reduce poverty of livestock keepers in developing countries through capacity building and enabling development, testing, increasing production, and delivery of surveillance, prevention, and control tools in disease-endemic communities such as a vaccine to prevent porcine cysticercosis.

Three working groups were formed during the meeting to consider priority issues relating to human and porcine infections as well as prevention and control tools needing attention. They were tasked with considering opportunities for more concerted efforts to address these needs and possibilities for mobilizing resources to enable these efforts as well as any other relevant issues. The working groups met during the meeting and then presented results of their deliberations during the final session.

## H3 Human Cysticercosis

### Taeniasis, and Epilepsy Working Group

This group identified a series of priority issues needing both policy and research attention. The first topice considered was the lack of a clear definition of the distribution of taeniasis and human cysticercosis and the need for identifying regions where porcine cysticercosis is prevalent. Baseline mapping of domestic pig distributions and density complemented by rapid assessments of cysticercosis prevalence in the different pig populations should be a priority. Ideally, butchered pigs/pork (e.g., at weekly markets) could be used as sentinels for the prevalence in the surrounding landscape. After areas where the parasites circulate in pigs are identified, community-based epidemiologic studies should be undertaken using computed tomography (where available), serologic tests, or both, to assess the scale of human taeniosis and cysticercosis/nonsymptomatic neurocysticercosis in the community. Reducing infection rates in human carriers of adult tapeworms is ultimately the best way to reduce environmental contamination.

## H3 Porcine Cysticercosis

### Veterinary Public Health, and Tropical Pig Husbandry Working Group

The group recognized that poor veterinary public health infrastructure and lack of proper meat inspection and control are major factors that enable transmission. The group also agreed that if infected pig carcasses are totally condemned, a strong incentive exists for pig keepers to slaughter their pigs and sell the meat clandestinely. A trace-back system would assist with surveillance, for example*.* identifying disease-endemic hot spots. Investigating methods for safely processing cyst-infected meat under local conditions to maintain a minimum value would also be helpful. More attention should be given to understanding the importance of pigs to rural households and the different management systems being practiced.

## H3 Tools for Surveillance

### Prevention, and Control Working Group

The strong interest and expertise in diagnostics and vaccines in the European region were noted. Priority issues identified were improving the antibody and antigen assays and modifying them into more practical formats, e.g., dipsticks and lateral flow tests for field use in disease-endemic areas, as well as standardizing them at the global level. The candidate cysticercosis vaccines for pigs based on oncosphere antigens, for example, TSOL18/HP6-Tsol, show great promise for blocking transmission to humans. Further research is needed to ensure effective field application of the vaccines with regard to efficacy, delivery, duration of effect, and market acceptability.

## H3 Conclusion

The meeting was an effective way to enable knowledge sharing and promote a regional concerted effort for combating cysticercosis. Attendees will constitute the new Cysticercosis Working Group in Europe and will continue meeting every 12–18 months under the auspices of the Global Campaign for Combating Cysticercosis.

